# Untargeted GC–MS metabolic profiling of *Eurotium chevalieri* AUMC 16390 (PX498623) reveals a putative steroidal metabolite with antibacterial and anti-inflammatory potential

**DOI:** 10.1186/s12896-026-01104-6

**Published:** 2026-02-21

**Authors:** O. M. O. El-Maghraby, Kaoud Salama, M. S. Youssef, M. Marwa Abdel-Kareem, A. Randa Fathy

**Affiliations:** 1 Department of Botany and Microbiology, Faculty of Science, University of Sohag, Sohag, Egypt; 2 Czech Advanced Technology and Research Institute (CATRIN) & Institute of Molecular and Translational Medicine (IMTM), Palacký University Olomouc, Olomouc, Czech Republic; 3 Department of Chemistry, Faculty of Science, University of Sohag, Sohag, Egypt

**Keywords:** *Eurotium chevalieri*, GC–MS, Antimicrobial activity, FabI inhibitors, Putative natural steroid

## Abstract

**Background:**

This study investigated the exometabolome bioactive metabolites produced by *Eurotium chevalieri* AUMC 16,390 (accession number PX498623), a strain identified through ITS rDNA sequencing with 100% identity. The aim was to characterize its chemical profile and evaluate the biological activities of its major constituents.

**Materials and methods:**

Metabolites present in the crude extract were analyzed using GC–MS, and ten major compounds were detected, spanning esters, diketones, terpenoids, and halogenated hydrocarbons. A predominant putative steroid-like metabolite, tentatively identified as 12-hydroxy-(5α,12β)-androstane-3,17-dione, accounted for 35.79% of the extract. Antibacterial activity of the crude extract was assessed against multiple bacterial strains. To elucidate potential mechanisms, molecular docking studies were conducted targeting enoyl-acyl carrier protein reductase (FabI). Additionally, the anti-inflammatory potential of the major steroidal compound was examined via predicted interactions with the glucocorticoid receptor (GR). Physicochemical and pharmacokinetic properties were evaluated using SwissADME.

**Results:**

The crude extract demonstrated broad-spectrum antibacterial activity. Docking analysis revealed favorable binding affinities of the major steroid-like metabolite toward FabI, supporting its potential antibacterial mechanism. The compound also showed high predicted affinity for the GR, suggesting possible anti-inflammatory activity. SwissADME results indicated acceptable drug-likeness features and favorable oral bioavailability parameters.

**Conclusion:**

*Eurotium chevalieri* AUMC 16,390 represents a promising source of bioactive fungal metabolites. The major putative steroidal component exhibits strong potential as an antimicrobial and anti-inflammatory agent, providing a foundation for future experimental validation and development.

**Graphical Abstract:**

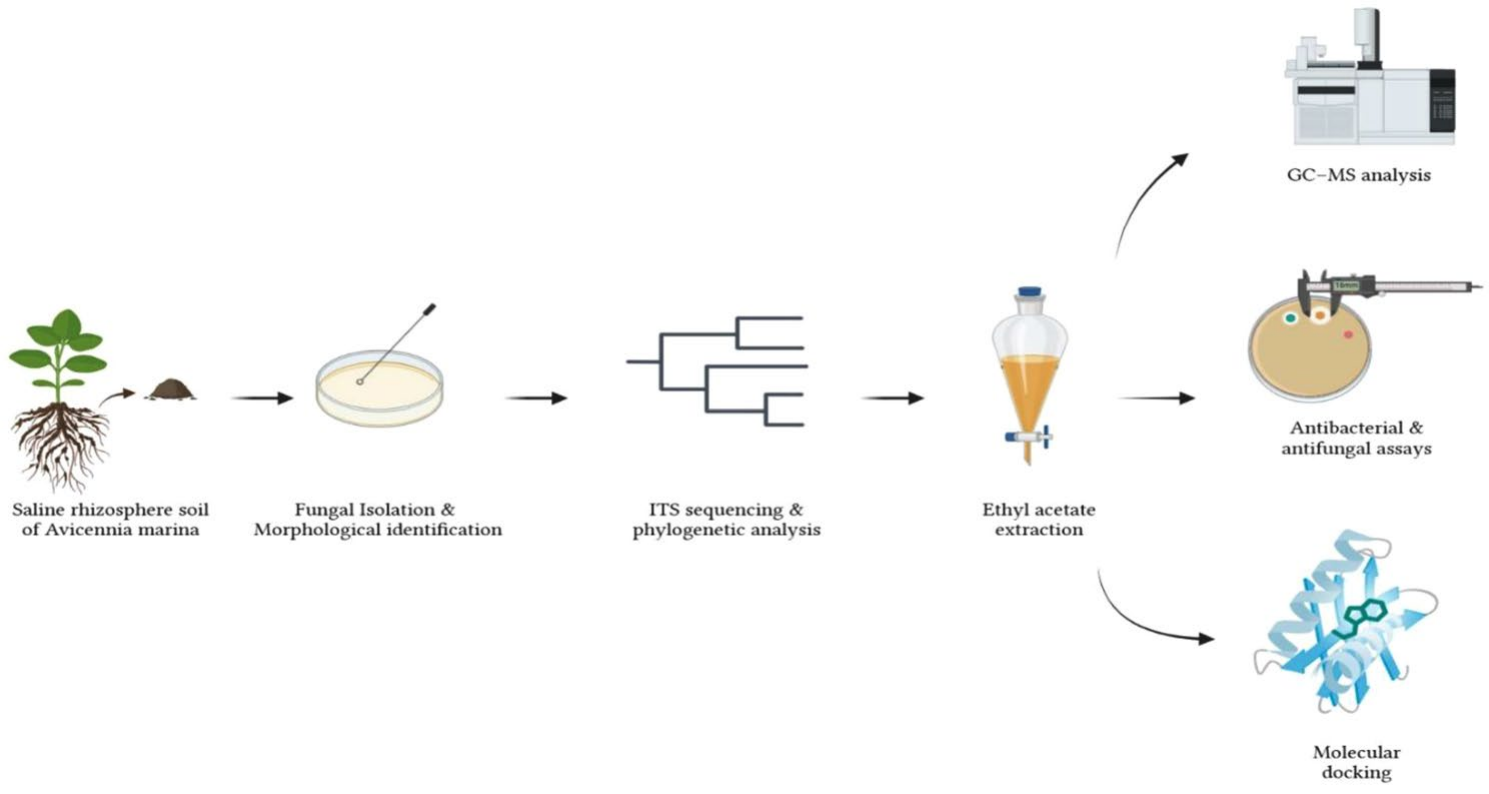

## Introduction

The abundance of structurally complex and physiologically active chemicals found in fungal secondary metabolites has greatly aided in the development of medicinal medicines, especially antibiotics and immunomodulators [1, 2]. Despite earlier underestimations of the metabolic variety of filamentous fungi, advances in molecular biology and analytical techniques have uncovered their remarkable biosynthetic capabilities and chemical diversity [3]. Because these metabolites are frequently employed as defense chemicals and ecological mediators, they are valuable for drug development and biochemical study [4].

Alkaloids, steroid-like chemicals, anthraquinones, and derivatives of benzaldehyde are some of the chemical classes that include fungal secondary metabolites. Many of these substances have cytotoxic, antioxidant, antibacterial, anticancer, and anti-inflammatory properties [5]. Eurotium species (teleomorph of Aspergillus) are known to produce bioactive metabolites, and some substances produced from Eurotium spp. have shown promising pharmacological properties. Asperflavin extracted from E. amstelodami, for instance, has been shown to have anti-inflammatory properties by preventing LPS-stimulated macrophages from producing prostaglandin E2 and nitric oxide without causing harm to cells [6].

Approximately 50% of approved medications are derived from or based on natural ingredients. This demonstrates that a significant portion of contemporary medications still come from natural sources [5, 6]. Given this, investigating understudied fungal taxa, such as Eurotium species, is still a feasible strategy for finding new bioactive substances [7]. The metabolic profile and biological potential of Eurotium chevalieri are still little understood, despite growing interest in this genus.

In particular, little is known about the steroid-like metabolites produced by Eurotium chevalieri, and their potential roles in antibacterial and anti-inflammatory properties have not been well investigated [8–10]. It is still believed that fungi constitute a rich source of secondary metabolites with a variety of structural variations and a broad range of pharmacological and biological effects [11]. Recent thorough reviews of fungal natural products have demonstrated that these contain terpenoids, alkaloids, polyketides, steroids, and other natural products of therapeutic significance [12]. Even though GC-MS-based metabolite profiling makes it easy to quickly and non-specifically identify the main fungal components, these techniques are usually used alone without including further predictive studies [13]. When it is not feasible to isolate compounds and immediately confirm their structure, metabolite profiling combined with molecular docking, cheminformatics, and metabolomics-guided approaches provides a helpful method for developing mechanistic hypotheses that link chemical composition to biological effects [14]. In order to link chemical variety with possible bioactivity and therapeutic importance, this integrated approach has become more popular in recent fungal natural product research.

In this work, Eurotium chevalieri will be identified using morphological and phylogenetic methods, its main extracellular metabolites will be characterized using GC-MS analysis, and the antimicrobial and possible anti-inflammatory qualities of these metabolites will be evaluated using molecular docking against FabI and the glucocorticoid receptor. Particular focus is paid to a steroid-like substance that was shown to be a significant component of the extract. Using retention index analysis and mass spectrum matching, this chemical is tentatively identified.

## Material and methods

### Isolation, purification, and identification of *Eurotium chevalieri*

The *E. chevalieri* isolate, a fungus capable of tolerating or thriving in high-salinity environments, was obtained from saline soils in the rhizosphere of *Avicennia marina* (a dominant mangrove species) along the Red Sea Coast of Upper Egypt (Red Sea Governorate), using Czapek’s agar supplemented with 5% NaCl [15]. Preliminary identification was conducted based on its morphological characteristics [16, 17] when cultivated on Czapek’s agar enriched with 10% sucrose at 28 ± 1 °C.

### Growth of the isolate and phylogenetic analysis of *Eurotium chevalieri*

The isolate was cultivated on CYA plates and maintained at a temperature of 25 ± 1 °C for 7 days [18]. A small quantity of fungal mycelium was scraped and suspended in 100 microliters of distilled water, then boiled at 100 degrees Celsius for 15 minutes before being stored at −70 degrees Celsius. The sample was forwarded to SolGent Company in Daejeon, South Korea, to undertake the entire process, beginning with DNA extraction and concluding with DNA sequencing. The CLCBio Main Workbench program was utilised to generate contigs from the sequencing data. The sequences obtained were further analyzed using the BLAST tool on the National Center for Biotechnology Information website. Sequences acquired along with those retrieved from the GenBank database (http://www.ncbi.nlm.nih.gov) were analyzed using Clustal W with MegAlign software, version 5.05, developed by DNASTAR Inc. of Madison, Wisconsin (USA), for phylogenetic purposes [19]. Phylogenetic relationships were inferred using the neighbor-joining (NJ) method based on Kimura’s two-parameter model.

### Subculture, inoculation of *Eurotium chevalieri,* and extraction of metabolites

*Eurotium chevalieri* (AUMC 16,390) was initially cultured on 10% sucrose–Czapek’s agar to obtain sufficient conidial and/or hyphal biomass following incubation for 9 days at 28 ± 1 °C. The fungus was then cultivated under static conditions in 50 ml of 3% sucrose–Czapek’s broth supplemented with peptone and yeast extract (1 g/l each) and trace elements (FeSO₄, MnSO₄, and ZnSO₄; 100 ppm each) at 28 ± 1 °C for 15 days.

After incubation, the culture broth was separated from the fungal biomass by filtration through Whatman No. 1 filter paper. The culture broth was filtered through sterile Whatman No. 1 filter paper under aseptic conditions. To confirm the sterility of the filtrate and the absence of residual fungal spores or hyphae, aliquots of the filtrate were plated onto potato dextrose agar and incubated at 28 °C for 72 h. No fungal growth was observed, confirming the sterility of the filtrate prior to extraction. The cell-free filtrate was stored at − 20 to −22 °C prior to antimicrobial evaluation using the agar well diffusion method. For metabolite extraction intended for GC–MS analysis and agar disc diffusion assays, ethyl acetate (75 mL) was added directly to the culture broth, and the mixture was shaken at 120 rpm overnight. This extraction step was performed twice to ensure efficient recovery of extracellular metabolites.

The combined ethyl acetate extracts were filtered through Whatman No. 1 filter paper, dried over anhydrous sodium sulfate (Na₂SO₄), and concentrated under reduced pressure. The resulting crude extracellular extract was stored at − 70 °C until further analysis by GC–MS and antimicrobial testing using the agar disc diffusion method.

### Preparation of extract for GC-MS analysis

The crude extract dissolved in ethyl acetate (1 ml) and 20 μl of diluted crude of the fungus (*Eurotium chevalieri* AUMC 16,390) was employed for GC/MS analysis.

### Instruments and chromatographic conditions

A Perkin Elmer Clarus 500 system with a gas chromatograph, an autosampler (AOC-20i), and a quadrupole mass spectrometer running in electron impact (EI) mode at 70 eV was used for low-resolution gas chromatography–mass spectrometry (GC–MS). A 100% dimethyl polysiloxane Elite-1 fused silica capillary column (30 m × 0.25 mm ID × 1 μm film thickness) was used to accomplish separation. The carrier gas was helium (99.999%) at a steady flow rate of 1 mL/min. The ion source temperature was 280 °C, and the injector temperature was set at 250 °C with a split ratio of 10:1. The oven temperature was set at 110 °C isothermal for two minutes, then ramped up to 200 °C at a rate of 10 °C per minute, 280 °C at a rate of 5 °C per minute, and maintained at 280 °C for nine minutes. Mass spectra were recorded over a range of m/z 40–550 with a scan interval of 0.5 s.

### Retention index (RI) determination

The retention indices of detected compounds were calculated using a homologous series of n-alkanes (C8–C40) analysed under the same chromatographic conditions. RIs were calculated according to the Van den Dool and Kratz equation:

$$RI = 100 \times \left[ {n{\rm{ + }}{{{t_r}\left( {{\rm{compound}}} \right) - {t_r}\left( n \right)} \over {{t_r}\left( {n + 1} \right) - {t_r}\left( n \right)}}} \right]$$

where ${t_r}\left( {{\rm{compound}}} \right)$is the retention time of the compound, and ${t_r}\left( n \right)$ and ${t_r}\left( {n + 1} \right)$are the retention times of the n-alkanes eluting immediately before and after the compound. The calculated RIs were compared with literature values and NIST 14 library entries to support tentative compound identification.

The National Institute of Standards and Technology (NIST) database, which contains more than 62,000 reference spectra, was used to interpret the mass spectra of the compounds. Each compound was compared with known spectra in the NIST library. Identification was based on a combination of mass spectral similarity and retention index agreement, allowing tentative assignment of molecular weights and structural features. All metabolites, including the steroid-like compound, are reported as putative, pending confirmation by complementary techniques such as LC–MS in positive and negative ESI modes, HR-MS, and NMR spectroscopy.

A summary of the putative metabolites, their relative abundance, retention times, calculated RIs, and comparison with literature RIs is provided in Table 1.

**Table 1:** The dominant compounds (chemical structures and compositions), molecular formula, retention time (RT) min., concentrations (% per crude extract), molecular weights (M.W.) g/mol and retention index (RI) of *E. chevalieri* AUMC16390

No. of Compound	Chemical structure	Molecular Formula	M.W. (g/mol)	% of Compound in Extract	Retention time (min.)	Retention Index
1	Pentafluoropropionic acid, pentadecyl ester	C_18_H_31_F_5_O_2_	374.43	0.480	31.58	1824
2	(12bS)-(+)-8,11-Bihydroxy-12b-methyl-1 H-benzo [6, 7]phenanthro[10,1-bc]furan	C_19_H_12_O_3_	288.30	1.362	37.893	1973
3	5-Eicosene	C_20_H_40_	280.53	1.046	22.336	1561
4	12-Hydroxy-, (5.alpha. 12.beta.)-Androstane-3,17-dione	C_19_H_28_O_3_	304.42	35.788	34.555	1898
5	7,7,8,8-Tetramethylbicyclo[4.2.0]octa-1(6),3-diene-2,5-dione	C_12_H_14_O_2_	190.24	37.504	33.546	1873
6	Di-tert-Butylphenol	C_14_H_22_O	206.32	0.419	15.745	1326
7	(15E)-15-Heptadecenal	C_17_H_32_O	252.44	1.393	25.266	1651
8	Heptafluorobutyric acid, pentadecyl ester	C_19_H_31_F_7_O_2_	424.44	0.801	27.744	1722
9	Pentafluoropropionic acid, dodecyl ester	C_15_H_25_F_5_O_2_	332.35	0.392	17.077	1379
10	Phthalic acid, bis(2-ethylhexyl) ester	C_24_H_38_O_4_	390.56	19.179	34.361	1893

### Pre-germination of pathogenic micro-organisms (bacteria and fungi)

Bacterial isolates were obtained from the American Type Culture Collection (*Staphylococcus aureus* ATCC 25,923*, Bacillus cereus* ATCC 14,579 and *Bacillus subtilis* ATCC 6633, Gram +ve and *Escherichia coli* ATCC 25,922, *Klebsiella pneumoniae* ATCC 13,883, *Salmonella enterica* ATCC 14,028 and *Pseudomonas aeruginosa* ATCC 27,853, Gram –ve), were pre-germinated in slants of nutrient agar (peptone, 5 g/l, beef extract, 3 g/l, sodium chloride, 8 g/l and agar, 15 g/l), pH 7.3 ± 0.2, dispersed in 1 liter of dist. H_2_O, allow to soak for 10 minutes to mix well, then sterilize by autoclaving for 15 minutes at 121 °C. Whereas, pathogenic fungi were obtained from Assiut University (*Candida tropicalis* AUMC 9158 & *Candida albicans* AUMC 9138), were pre-germinated on Sabouraud’s agar (dextrose, 40 g/l, peptone, 10 g/l and agar,15 g/l), pH 5.6 ± 0.2, at 37°Cfor 24 and 48 h, respectively, to have a mass of spores or conidia. Bacterial inocula were prepared by suspending freshly grown colonies in sterile saline solution and adjusting turbidity to 0.5 McFarland standard, corresponding to approximately 1 × 10^8^ CFU/mL. Fungal inocula were prepared using spore suspensions adjusted to approximately 1 × 10^6^ spores/mL, as determined by hemocytometer counting. After the incubation periods ended, 5 ml of sterilized distilled water was added to the microorganism culture, smoothed out with a loop, and then transferred to a 50 ml Erlenmeyer flask containing 20 ml of sterilized distilled water. It was then hand-shaken and directly used for inoculating the media in a Petri dish.

### Determination of anti-bacteria and anti-fungi test

Agar well and disc diffusing methods were used, where thick layers (~5 mm) of appreciated media (nutrients and Sabouraud’s agar) for bacterial and fungal growth were done in Petri dishes (9 or 11 cm^2^). The media were separately inoculated with the desirable organism (1 ml) and dispersed on the surface of the solidified medium using a loop. Holes were made using a sterilized cork borer (1 cm^2^), and the filtered broth media (each, 0.5 ml) of *Eurotium chevalieri* AUMC 16,390 was poured into the holes. Also, discs of filter paper (5 mm) were separately saturated with the crude extract (ethyl acetate), dried and fixed onto the tested media of bacteria and fungi. The dishes were incubated at the optimum temperature for the growth of the organism as previously described. After incubation periods had ended, the average of the two diameters was calculated and served as a measure of the filtered broStandardized paper disks saturated with crude extract were placed onto the agar surface that had been inoculated.oculated agar surface. Following this, the Petri dish was incubated at 37 °C for 24–48 hours, and the diameter of the inhibition zone was then measured. Ethyl acetate was used as the solvent for dissolving the fungal crude extract. A negative control consisting of pure ethyl acetate was included in all antimicrobial assays to exclude any potential inhibitory effects of the solvent. Standard antibiotics were used as positive controls to validate the antimicrobial assay. Cefotaxime was employed as positive controls for Gram-positive and Gram-negative bacteria, while Fluconazole was used as a positive control for antifungal activity. All controls were tested under identical conditions to those used for the fungal crude extract. All antimicrobial assays were performed in triplicate, and inhibition zone diameters were measured in millimeters. Results are presented as mean ± standard deviation (SD).

### Statistical analysis

Antimicrobial activity data were obtained from triplicate biological replicates and are presented as mean values ± SD. Statistical evaluation was limited to descriptive statistics due to the preliminary nature of the study.

### Molecular modeling

Before docking, the identified secondary metabolites were energy-minimized using Open Babel implemented in PyRx with the Universal Force Field (UFF) and a conjugate gradient algorithm (200 steps). The optimized ligand structures were converted into PDBQT format for docking analysis.

The crystal structures of Escherichia coli enoyl-acyl carrier protein reductase (FabI) (PDB ID: 1QG6, 1.80 Å) and the glucocorticoid receptor (GR) (PDB ID: 1M2Z, 2.5 Å) were retrieved from the Protein Data Bank (PDB). Protein preparation was carried out using AutoDock Tools, where co-crystallized ligands, water molecules, and ions were removed, followed by the addition of polar hydrogen atoms and assignment of Kollman charges. The prepared protein structures were saved in PDBQT format.

Molecular docking simulations were performed using AutoDock Vina, as implemented in PyRx. For FabI, the docking grid was defined based on the binding site of the co-crystallized ligand triclosan, with the grid box centered at × = 12.45, y = −6.82, z = 18.31 and dimensions of 22 × 22 × 22 Å, adequately covering the catalytic pocket. For the glucocorticoid receptor, the docking grid was centered on the ligand-binding domain using the coordinates of the co-crystallized ligand dexamethasone. An exhaustiveness value of 8 was applied in all docking calculations to ensure sufficient conformational sampling.

To validate the docking protocol, re-docking of the co-crystallized ligands was performed. Triclosan was re-docked into the FabI active site, and dexamethasone was re-docked into the ligand-binding pocket of GR. Docking accuracy was evaluated by calculating the root-mean-square deviation (RMSD) between the re-docked poses and their corresponding crystallographic conformations. In both cases, the obtained RMSD values were below 2.0 Å, confirming the reliability and reproducibility of the docking procedure for both targets.

The resulting docked complexes were analyzed using PyMOL to visualize ligand–protein interactions, including hydrogen bonds and hydrophobic contacts. Docking scores generated by AutoDock Vina were used for comparative ranking of ligands, recognizing that the scoring function is empirical and associated with an estimated uncertainty of approximately ±2–3 kcal/mol, and thus not intended to represent absolute binding free energies.

### Drug-likeness and ADME profiling

The physicochemical and drug-likeness characteristics of the identified secondary metabolites were assessed using DataWarrior version 6.5, which is a product of Actelion Pharmaceuticals in Allschwil, Switzerland, and the SwissADME online platform [20]. Molecular descriptors were calculated from compounds submitted in SMILES format, encompassing properties such as molecular weight, molecular formula, hydrogen bonding capabilities, cLogP, cLogS, total surface area, polar surface area, and relative polar surface area. Drug-likeness scores were subsequently estimated in DataWarrior by assessing fragment contributions and deviations from established drug-like characteristics. SwissADME predictions concurrently offered insights into physicochemical properties, lipophilicity, solubility, pharmacokinetics, and the BOILED-Egg model for gastrointestinal absorption and brain penetration. Oral bioavailability-related features such as lipophilicity, size, polarity, solubility, flexibility, and saturation were visualized using radar plots. Results were tabulated and visualized after being exported, following analyses conducted under default settings.

## Results

### Identification of *Eurotium chevalieri* and its strain

**Fig. 1:**
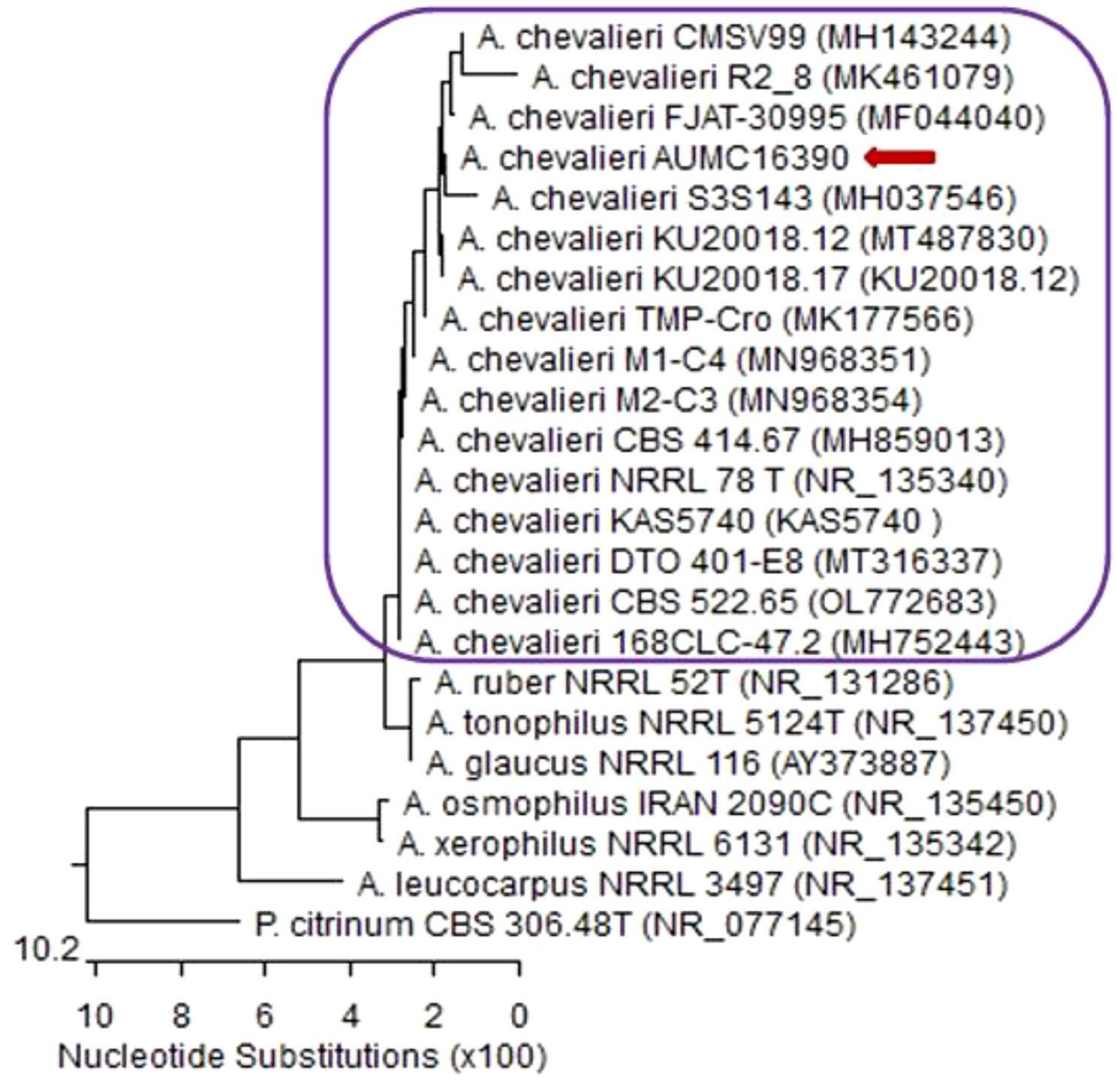
Phylogenetic tree of *E. chevalieri* (AUMC 16,390) based on ITS sequences of rDNA

Based on the two identification methods (morphological characteristics and phylogenetic analysis, Fig. 1), the isolate was identified as *Eurotium chevalieri* and listed in Assiut University Mycological Center as strain *Eurotium chevalieri* AUMC 16,390, which is used in the following study. It is worth mentioning that ITS sequences of rDNA aligned with closely related strains accessed from GenBank. The strain (*E. chevalieri* AUMC 16,390) showed 99.8% − 100% identity and 100% coverage with several strains of the same species (strains) as shown in Fig. 1. With internal transcribed spacer (ITS) sequences and accession number PX498623.

GGAAGGATCATTACCGAGTGCGGGCCCTCTGGGTCCAACCTCCCATCCGTGTCTATCTGTACCCTGTTGCTTCGGCGTGGCCACGGCCCGCCGGAGACTAACATTTGAACGCTGTCTGAAGTTTGCAGTCTGAGTTTTTAGTTAAACAATCGTTAAAACTTTCAACAACGGATCTCTTGGTTCCGGCATCGATGAAGAACGCAGCGAAATGCGATAATTAATGTGAATTGCAGAATTCAGTGAATCATCGAGTCTTTGAACGCACATTGCGCCCCCTGGTATTCCGGGGGGCATGCCTGTCCGAGCGTCATTGCTGCCCTCAAGCACGGCTTGTGTGTTGGGCTTCCGTCCCTGGCAACGGGGACGGGCCCAAAAGGCAGTGGCGGCACCATGTCTGGTCCTCGAGCGTATGGGGCTTTGTCACCCGCTCCCGTAGGTCCAGCTGGCAGCTAGCCTCGCAACCAATCTTTTTAACCAGGTTGACCTCGGATCAGGTAGGGATACCCGCTGAACTTAAGCATA

### GC-MS profile of *Eurotium chevalieri* AUMC 16,390 extract

**Fig. 2:**
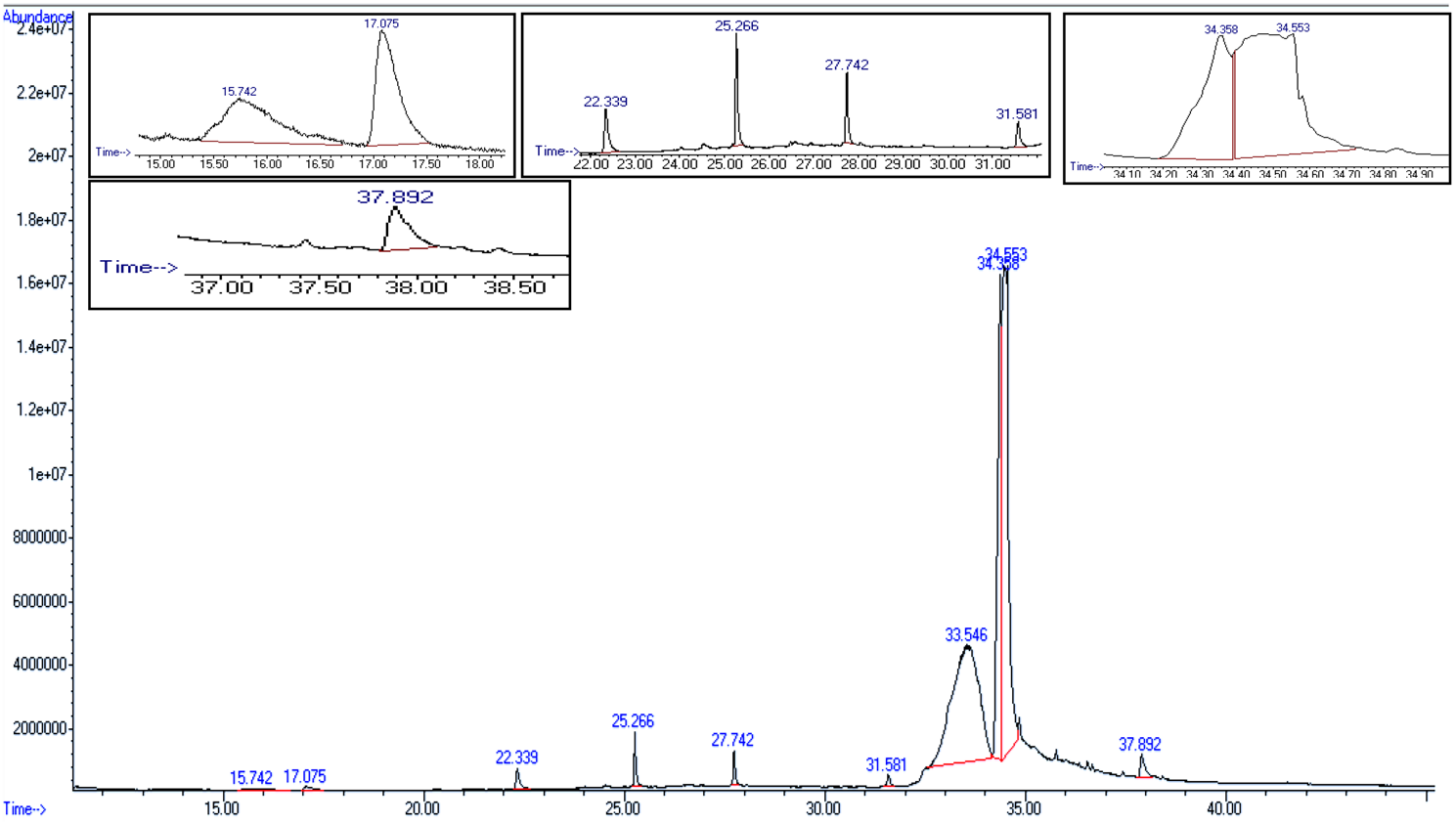
GC-MS chromatogram of ethyl acetate extract of *E. chevalieri* AUMC 16,390

The ethyl acetate extract of *Eurotium chevalieri* AUMC16390 was analyzed using gas chromatography–mass spectrometry (GC–MS) to characterize its dominant exometabolome metabolites (Fig. 2). The analysis revealed the presence of ten major compounds, accounting for 98.36% of the total extract, representing a chemically diverse metabolite profile (Table 1).

The most abundant constituents were 7,7,8,8-tetramethylbicyclo[4.2.0]octa-1(6),3-diene-2,5-dione (37.504%) and 12-hydroxy-(5α,12β)-androstane-3,17-dione (35.788%). Both compounds contain diketone functionalities, structural features frequently associated with biological activities such as cytotoxic and anti-inflammatory effects. In addition, phthalic acid, bis(2-ethylhexyl) ester constituted a substantial proportion of the extract (19.179%) and has been previously reported to exhibit antimicrobial and endocrine-disrupting properties.

Several fluorinated esters were detected in lower amounts, including pentafluoropropionic acid, pentadecyl ester (0.480%), heptafluorobutyric acid, pentadecyl ester (0.801%), and pentafluoropropionic acid, dodecyl ester (0.392%). The presence of halogenated moieties in these compounds suggests potential antimicrobial or oxidative stress-modulating activity. Other identified metabolites included (12bS)-(+)-8,11-bihydroxy-12b-methyl-1 H-benzo [6, 7]phenanthro[10,1-bc]furan (1.362%), a polycyclic oxygenated compound; 5-eicosene (1.046%), a long-chain alkene; (15E)-15-heptadecenal (1.393%), an unsaturated aldehyde; and di-tert-butylphenol (0.419%), a substituted phenol known for antioxidant potential.

Retention indices (RIs) were calculated using a homologous series of *n*-alkanes (C8–C40) analyzed under identical chromatographic conditions according to the Van den Dool and Kratz equation. The calculated RIs were compared with literature data and entries from the NIST 14 database to support compound identification. It should be emphasized that the structural assignments, particularly for complex metabolites such as the steroid-like compound, are considered tentative, as they are based on GC–MS spectral matching and RI comparison. Definitive structural confirmation will require further spectroscopic analyses, including high-resolution mass spectrometry (HRMS) and nuclear magnetic resonance (NMR) spectroscopy.

Overall, these results demonstrate the pronounced chemical diversity of *E. chevalieri* metabolites, encompassing ketones, esters, aldehydes, phenols, and halogenated derivatives. A comprehensive summary of the identified compounds, including their relative abundances, molecular formulas, molecular weights, retention times, and calculated retention indices, is presented in Table 1.

## Antimicrobial screening

The antimicrobial potential of the filtered broth medium and crude extract of (ethylacetate) *Eurotium chevalieri* AUMC 16,390 was evaluated against selected pathogenic microorganisms, including a bacterial species (*Staphylococcus aureus* ATCC 25,923*, Bacillus cereus* ATCC 14,579 and *Bacillus subtilis* ATCC 6633, Gram +ve and *Escherichia coli* ATCC 25,922, *Klebsiella pneumoniae* ATCC 13,883, *Salmonella enterica* ATCC 14,028 and *Pseudomonas aeruginosa* ATCC 27,853, Gram –ve) and two fungal strains (*Candida tropicalis* AUMC 9158 and *Candida albicans* AUMC 9138), using the agar well diffusion and disk diffusion methods (Table 2). The results revealed that Gram-negative bacteria exhibited higher sensitivity to both the filtered broth and crude extract, especially *E. coli* (33.43 ± 1 mm), compared to Gram-positive bacteria, as indicated by larger inhibition zones. In terms of antifungal activity, the crude extract of *E. chevalieri* showed inhibitory effects on both *Candida* species, producing inhibition zones of ~ 10 mm each. In contrast, the filtered broth medium demonstrated antimicrobial activity with an inhibition zone of ~ 16 mm. The ethyl acetate negative control showed no detectable inhibition zones against any of the tested microorganisms, confirming that the antimicrobial activity observed was due to the fungal crude extract. The positive controls exhibited pronounced inhibition zones against the tested microorganisms, confirming the suitability of the assay conditions. The antimicrobial activity of the fungal crude extract was evaluated relative to these standard drugs.

**Table 2:** Antimicrobial activity of *Eurotium chevalieri* using agar well and agar disc diffusion methods

Tested microorganism	Agar well-diffusing method (Inhibition zone) mm	Agar disc diffusing methods (Inhibition zone) mm	Positive control (mm)
**Pathogenic bacteria**			**Cefotaxime**
*Staphylococcus aureus* ATCC 25,923	12.15 ± 1.3	10.22 ± 0.5	31.3 ± 1.2
*Bacillus cereus* ATCC 14,579	16.10 ± 1.2	12.13 ± 0.8	35 ± 2
*Bacillus subtilis* ATCC 6633	16.16 ± 0.4	12.24 ± 0.6	39.33 ± 1.2
*Escherichia coli* ATCC 25,922	33.43 ± 1	25.37 ± 0.5	37.8 ± 0.6
*Klebsiella pneumoniae* ATCC 13,883	25.32 ± 1.4	20.11 ± 0.5	32.6 ± 0.6
*Salmonella enterica* ATCC 14,028	20.26 ± 0.6	16.29 ± 0.4	29.6 ± 0.6
*Pseudomonas aeruginosa* ATCC 27,853	25.10 ± 0.8	20.10 ± 0.2	30.3 ± 1.5
**Pathogenic fungi**			**Fluconazole**
*Candida tropicalis* AUMC 9158	15.60 ± 1.6	10.25 ± 0.2	19.96 ± 1.3
*Candida albicans* AUMC 9138	15.73 ± 0.6	10.45 ± 0.6	23.13 ± 0.6

## Secondary metabolites as inhibitors of enoyl-[acyl carrier protein] reductase (FabI)

To complement the experimental antimicrobial screening results obtained for our fungal metabolites, we conducted molecular docking of a focused library of 10 secondary metabolites against FabI from *Escherichia coli* (PDB ID: 1QG6) to assess their inhibitory potential. The active site was defined based on the location of the co-crystallized ligand, ensuring targeted docking with AutoDock Vina.

**Fig. 3:**
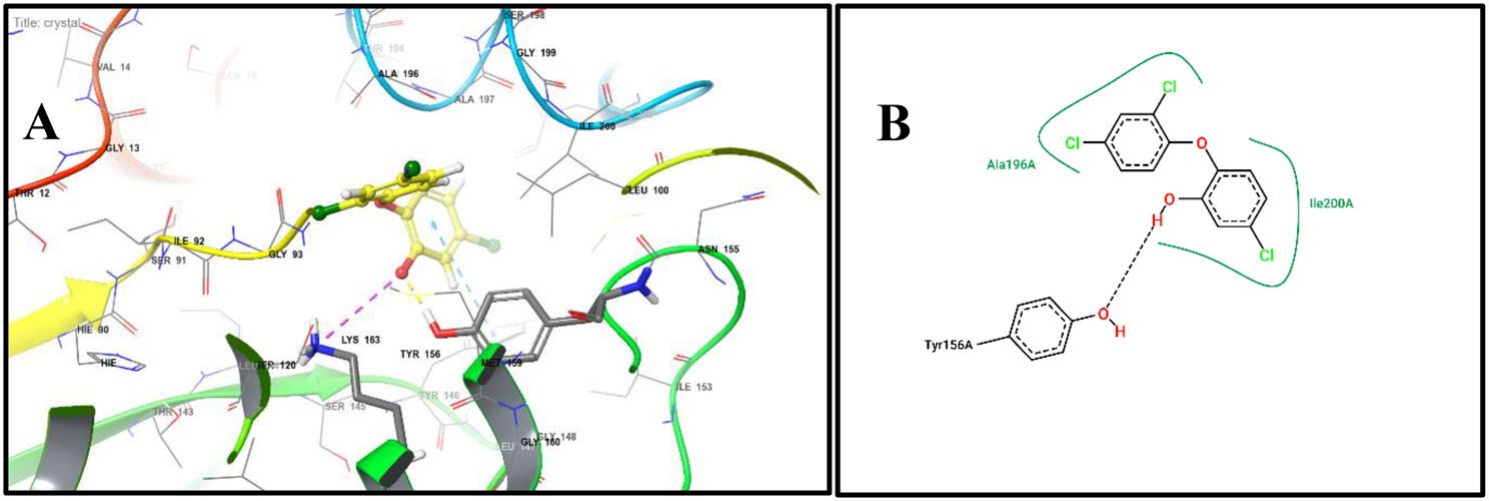
Redocking of triclosan in FabI : (**A**) 3D binding pose of the redocked co-crystallized ligand triclosan (yellow) within the active site of enoyl-[acyl carrier protein] reductase (FabI, PDB ID: 1QG6). Hydrogen bonds and hydrophobic interactions are highlighted. (**B**) 2D interaction diagram of triclosan showing key contacts with active site residues (Lys163A, Tyr156A, Thr194A,and Ile200A) essential for catalysis and inhibitor recognition, validating the docking protocol

The docking protocol was validated by re-docking the co-crystallized ligand, which reproduced binding affinities consistent with reported interactions, confirming the reliability of our approach (Fig. 3)

. Docking of the metabolites yielded binding affinities ranging from [−8.171 kcal/mol] to [−4.120 kcal/mol] (Table 3), with [4 out of 10] compounds showing scores equal to or better than the reference ligand [−6.517 kcal/mol], indicating promising activity.

**Table 3:** Binding affinity and molecular interactions of selected *E. chevalieri* metabolites with target protein

Comp.number	chemical structures and composition	Binding Affinity (kcal/mol)	H-Bond Interactions	Hydrophobic Interactions
1	(12bS)-(+)-8,11-Bihydroxy-12b-methyl-1 H-benzo [6, 7]phenanthro[10,1-bc]furan	−8.171	Gly93A	Ala196A Ile20A
2	Di-tert-Butylphenol	−6.924	Gly93A, Ile192A	Ile192A, Tyr146A
3	12-Hydroxy-, (5.alpha. 12.beta.)-Androstane-3,17-dione	−6.755	Lys163A	Ile200A, Ala197A, Ile20A
4	7,7,8,8-Tetramethylbicyclo[4.2.0]octa-1(6),3-diene-2,5-dione	−6.542	Tyr156A, Thr194A	Ile192A
5	Phthalic acid, bis(2-ethylhexyl) ester	−6.495	Tyr 156A	Ile200A, Met159A, Leu100A, Tyr146A, Ala196A, Ile20A
6	Pentafluoropropionic acid, pentadecyl ester	−6.140	Lys163A	Ile20A, Leu100A, Ala95A
7	Pentafluoropropionic acid, dodecyl ester	−6.140	Lys163A	Ile20A, Leu100A, Ala95A
8	Heptafluorobutyric acid, pentadecyl ester	−5.483	Lys163A	Ile20A, Leu100A, Ala95A
9	(15E)-15-Heptadecenal	−5.120	-	Met159A, Leu100A, Tyr146A, Ala196A, Ile20A
10	5-Eicosene	−4.120	-	Met159A, Leu100A, Tyr146A,

Detailed interaction analysis of the top-scoring metabolites revealed that they not only occupied the FabI binding pocket but also engaged key residues critical for enzyme inhibition.

The first compound,(12bS)-(+)-8,11-Bihydroxy-12b-methyl-1 H-benzo [6, 7]phenanthro- [10,1 bc]furan, (Fig.s 4A, B), formed hydrogen bonds with Gly93A, in addition to hydrophobic interactions with Ala196A and Ile20A in particular, has been reported to stabilize inhibitors through strong hydrogen bonding, highlighting the significance of this interaction.

**Fig. 4:**
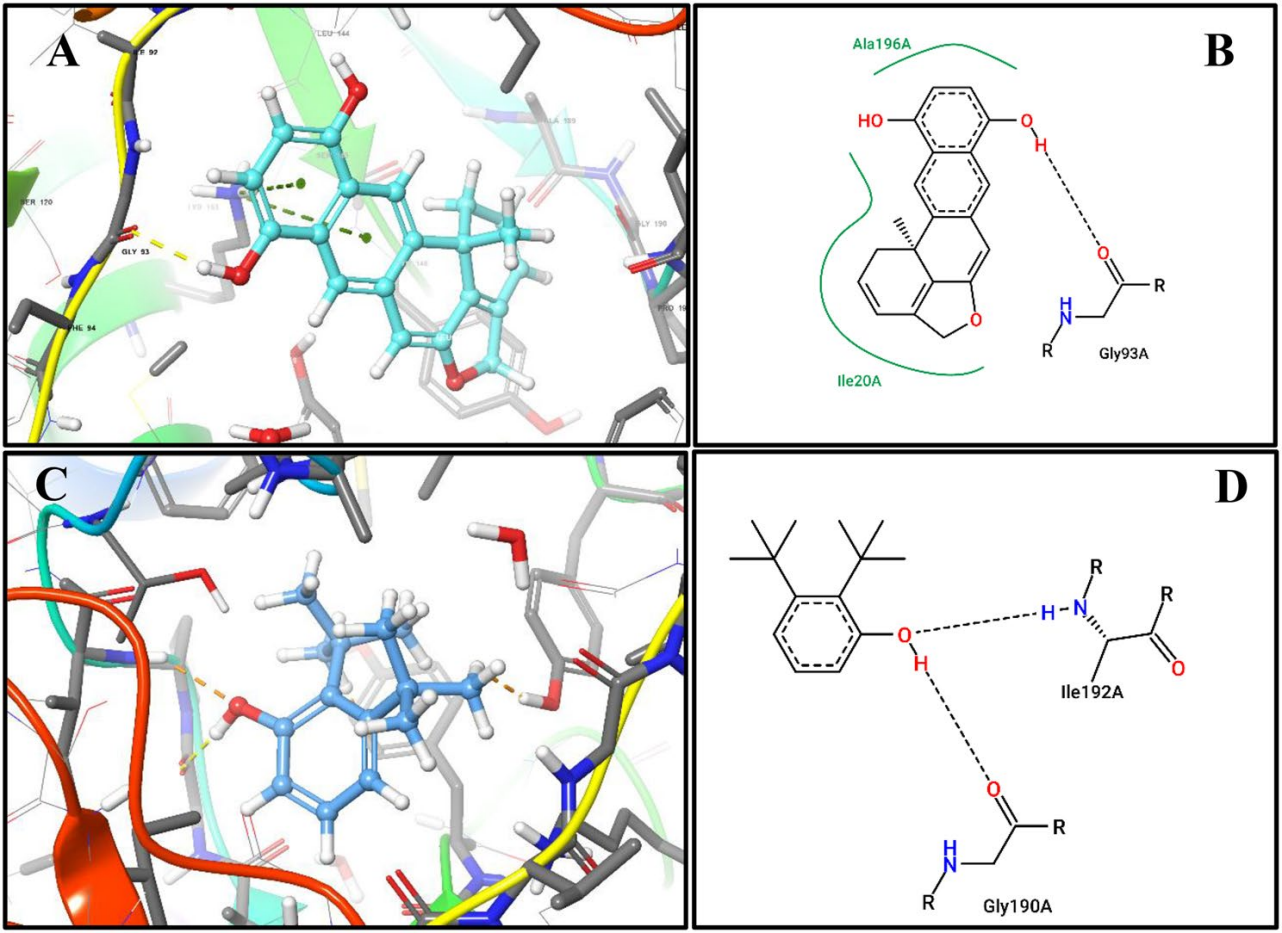
Docking of dominant FabI inhibitors from *E. chevalieri* extract: (**A**) 3D binding pose of compound 1 (cyan ball-and-stick) within the FabI active site. (**B**) 2D interaction diagram of compound 1 showing hydrogen bonds and hydrophobic contacts with FabI residues. (**C**) 3D binding pose of compound 2 (faded azure ball-and-stick) within the FabI active site. (**D**) 2D interaction diagram of compound 2 highlighting key interactions with FabI catalytic residues. *these docking results indicate potential inhibitory mechanisms of the compounds against FabI, correlating with antibacterial activity observed in the crude extract*

The second compound, Di-tert-Butylphenol (Fig 4C, D), exhibited hydrophobic contacts with Ile192A and Tyr146A, along with a hydrogen bond to Gly93A and Ile192A. This combination of polar and nonpolar interactions likely contributes to its enhanced docking score.

**Fig. 5:**
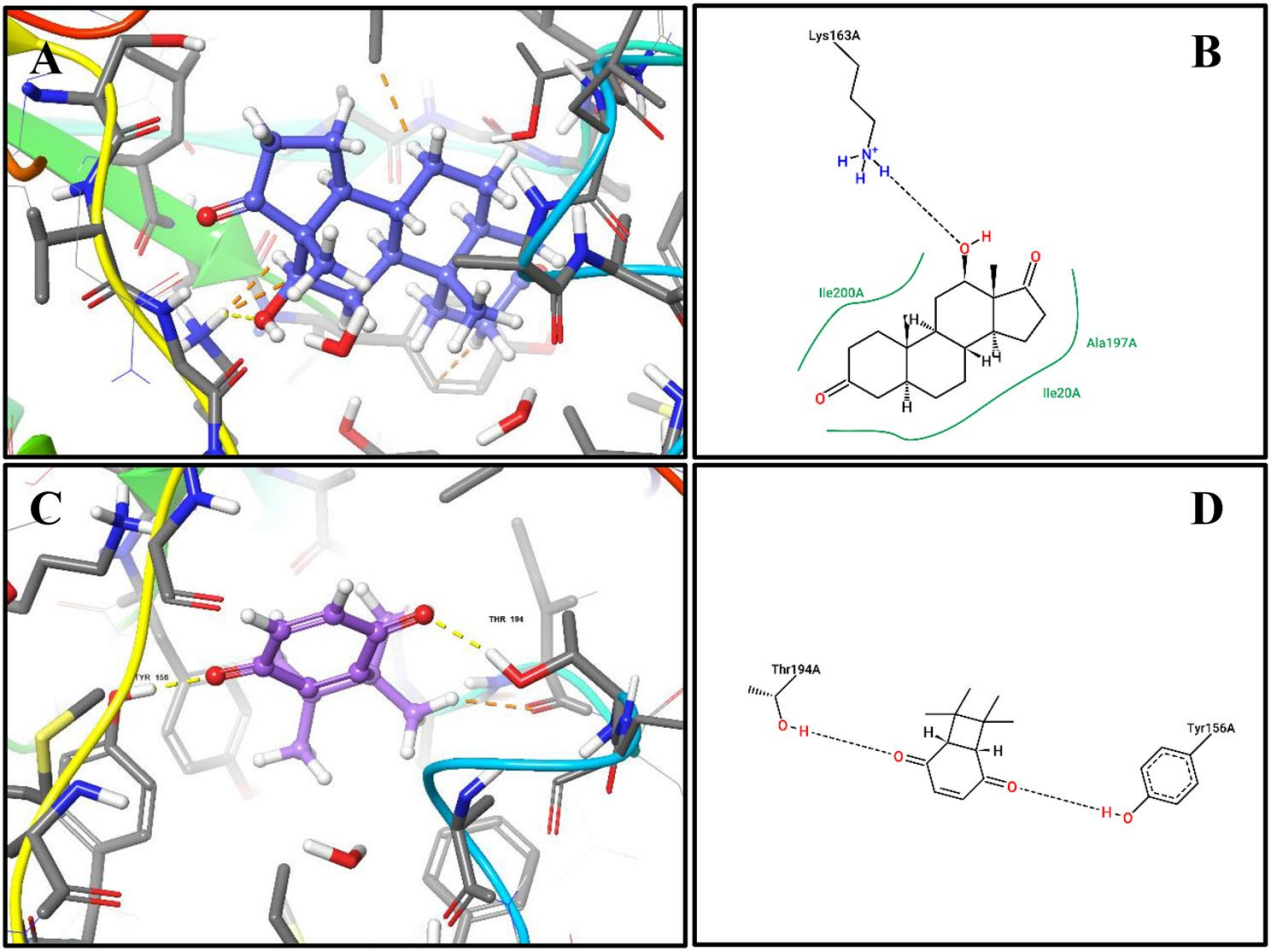
Docking of dominant FabI inhibitors from *E. chevalieri* extract: (A) 3D binding pose of compound 3 (blue ball-and-stick) within the FabI active site. (B) 2D interaction diagram of compound 3 showing hydrogen bonds and hydrophobic contacts with FabI residues. (C) 3D binding pose of compound 4 (violit ball-and-stick) within the FabI active site. (D) 2D interaction diagram of compound 4 highlighting key interactions with FabI catalytic residues. *these docking results indicate potential inhibitory mechanisms of the compounds against FabI, correlating with antibacterial activity observed in the crude extract*

The third compound, 12-Hydroxy-, (5.alpha. 12.beta.)-Androstane-3,17-dione, (Fig 5A, B) also showed a strong hydrogen bond with Lys163A, complemented by stabilizing hydrophobic contacts with Ile200A, Ala197A, and Ile20A, indicating optimal accommodation within the active site pocket.

The fourth compound, 7,7,8,8-Tetramethylbicyclo[4.2.0]octa-1 (6),3-diene-2,5-dione, (Fig.s 5C, D), also showed a strong hydrogen bond with Tyr156A and Thr194A, complemented by stabilizing hydrophobic contacts with Ile192A, indicating optimal accommodation within the active site pocket.

## Potential anti-inflammatory activity of the fungal steroid revealed by glucocorticoid receptor docking

Glucocorticoid receptor docking was selectively performed for Compound 3 (12-hydroxy-(5α,12β)-androstane-3,17-dione) based on structural considerations. GR is a steroid hormone receptor that preferentially binds ligands possessing a steroidal core framework. Among all identified metabolites, Compound 3 was the only compound exhibiting a cyclopentanoperhydrophenanthrene skeleton analogous to endogenous and synthetic glucocorticoids. Other major metabolites lacked structural features compatible with GR ligand recognition and were therefore not prioritized for GR docking analysis.

**Fig. 6:**
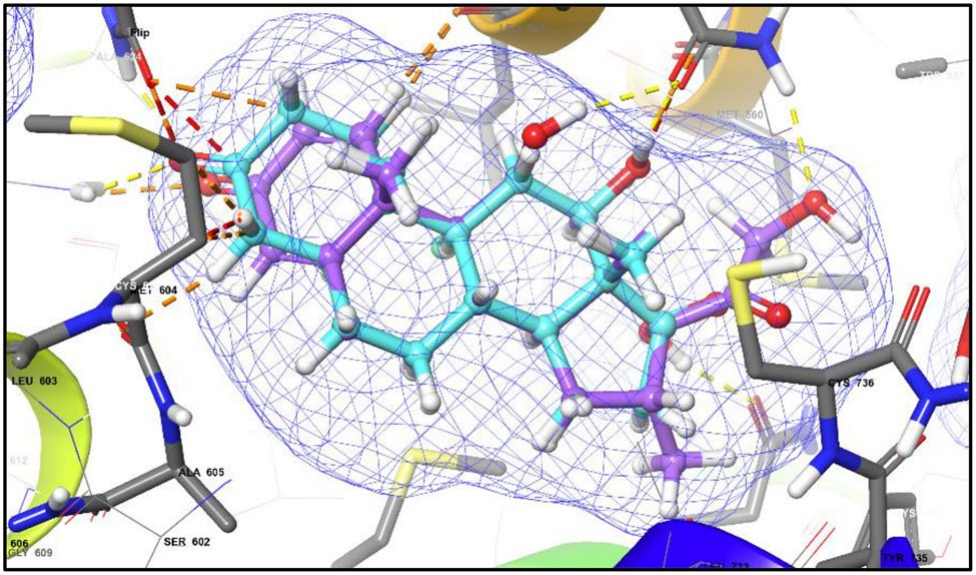
Superimposition of dexamethasone and the fungal steroid in GR

**Fig. 7:**
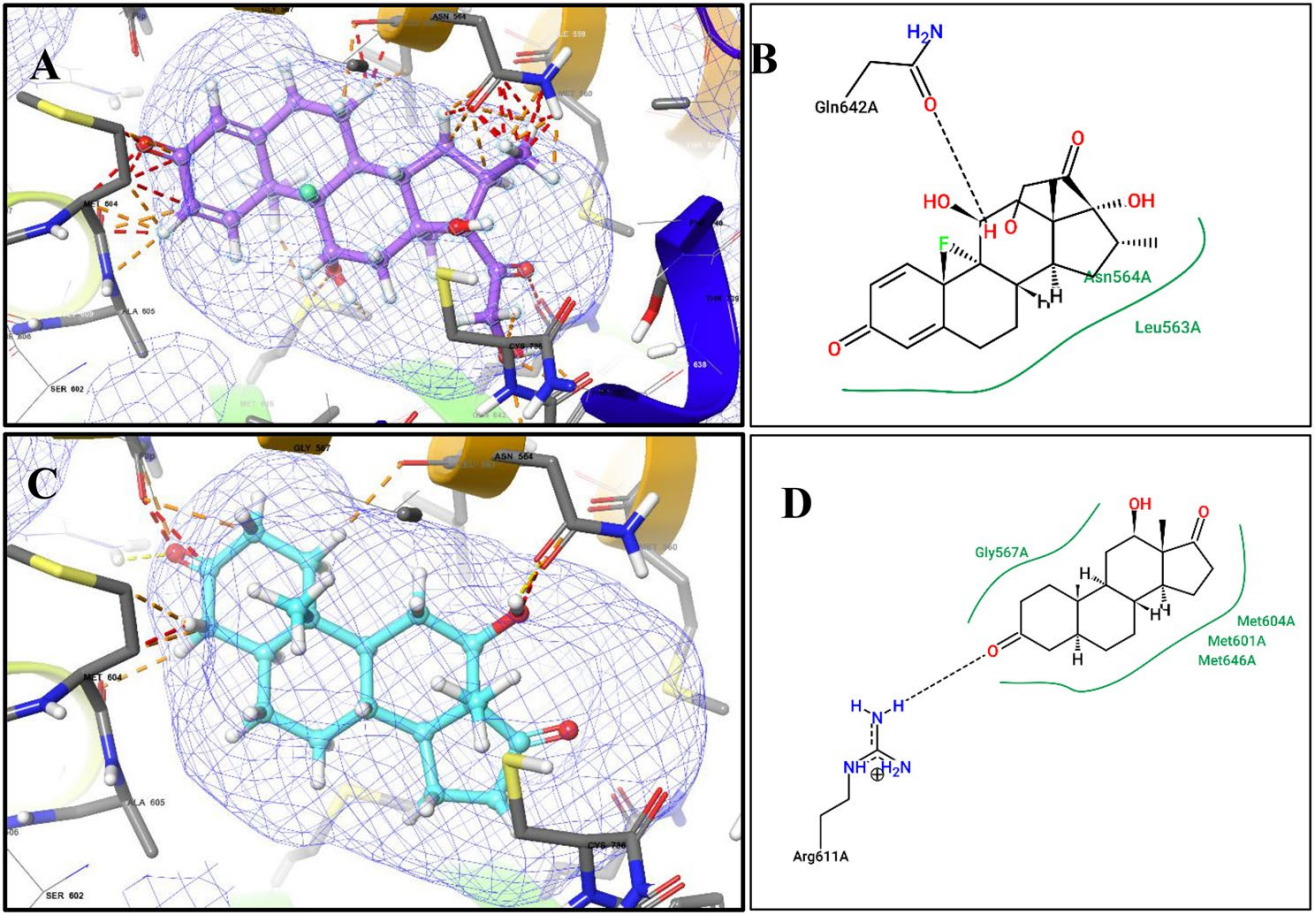
Docking of dexamethasone and the putative fungal steroid in the glucocorticoid receptor (GR):(**A**) 3D binding pose of dexamethasone (violet ball-and-stick) within the GR ligand-binding domain (PDB 1M2Z). (**B**) 2D interaction diagram of dexamethasone showing hydrogen bonds and hydrophobic interactions with GR residues (Arg611A, Met604A, Met646A) important for receptor activation. (**C**) 3D binding pose of 12-hydroxy-(5α,12β)-androstane-3,17-dione (cyan ball-and-stick) within the GR active site. (**D**) 2D interaction diagram showing key interactions with GR residues, providing a putative molecular basis for anti-inflammatory potential

Molecular docking of 12-Hydroxy-,(5α,12β)-Androstane-3,17-dione into the glucocorticoid receptor (PDB ID: 1M2Z) demonstrated a favourable binding profile, with a docking score of −10.271 kcal/mol, comparable to that of the reference ligand dexamethasone (−12.06 kcal/mol). Superimposition of the fungal steroid with dexamethasone yielded an RMSD of 2.085 Å, indicating a similar orientation within the GR binding pocket and the potential to mimic key ligand–receptor interactions (Fig. 6). Dexamethasone forms hydrogen bonding with Gln642A and hydrophobic contact with Leu563A (Fig. 6A,B), whereas 12-Hydroxy-,(5α,12β)-Androstane-3,17-dione interacts through hydrogen bonding with Arg611A and hydrophobic contacts with Gly567A, Met604A, and Met646A (Fig. 7C,D). These interactions underline the ability of the fungal steroid to establish stable binding within the GR active site and potentially elicit comparable biological activity.

Overlay of dexamethasone (cyan) and 12-hydroxy-(5α,12β)-androstane-3,17-dione (magenta) within the GR binding pocket. The RMSD value of 2.0 Å indicates similar binding orientation and conservation of key hydrogen bond and hydrophobic interactions, supporting the proposed receptor engagement of the fungal steroid.

## ADME properties

Physicochemical analysis (Table 4) revealed a cLogP of 2.89, reflecting moderate lipophilicity favourable for membrane permeability. The cLogS of −3.98 indicates moderate solubility, suitable for oral administration. The compound possesses 3 hydrogen bond acceptors and 1 hydrogen bond donor, providing an appropriate balance between polarity and hydrophobicity for receptor binding. The total surface area (219.27 Å^2^) and polar surface area (54.37 Å^2^) are consistent with compounds likely to be well absorbed and permeable, while the relative PSA (0.178) supports its ability to cross biological membranes. Importantly, the molecule satisfies drug-likeness criteria according to Lipinski, Ghose, Veber, Egan, and Muegge rules, highlighting its potential as an orally active agent.

**Table 4:** Drug-likeness and physicochemical properties of 12-hydroxy-(5α,12β)-androstane-3,17-dione based on in silico ADME analysis

cLogP	cLogS	H-Acceptors	H-Donors	Total Surface Area	Relative PSA	Polar Surface Area	Drug-likeness (Lipinski, Ghose, Veber, Egan, and Muegge rule)
2.8923	−3.977	3	1	219.27	0.17868	54.37	Yes

**Fig. 8:**
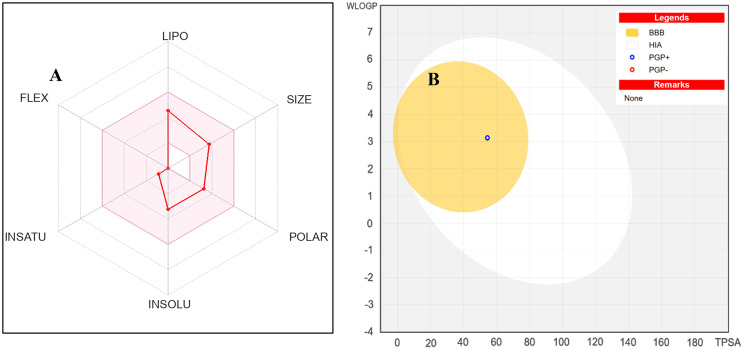
Predicted oral bioavailability and pharmacokinetic properties of the fungal steroid

Further evaluation of drug-likeness and pharmacokinetic behaviour using SwissADME supported these findings. The bioavailability radar showed that all six parameters—lipophilicity, size, polarity, solubility, saturation, and flexibility—fall within the optimal physicochemical range for oral bioavailability, indicating a balanced profile with good membrane permeability and suitable rigidity typical of steroidal scaffolds (Fig. 8A). Additionally, the BOILED-Egg model predicted efficient human intestinal absorption (HIA) and possible blood–brain barrier (BBB) penetration, consistent with its moderate polarity and lipophilicity (Fig. 8B). The compound was classified as a P-glycoprotein (PGP+) substrate, suggesting active efflux that could limit central nervous system accumulation, a desirable feature for minimizing corticosteroid-like side effects.

Taken together, the docking results and pharmacokinetic predictions suggest that 12-Hydroxy-,(5α,12β)-Androstane-3,17-dione is a promising natural steroidal scaffold for the development of anti-inflammatory agents, meriting further in vitro and in vivo evaluation.

(A) Bioavailability radar for 12-hydroxy-(5α,12β)-androstane-3,17-dione generated using SwissADME. The radar summarizes six physicochemical properties (lipophilicity, size, polarity, solubility, saturation, and flexibility) within optimal ranges for oral bioavailability.
(B) BOILED-Egg model predicting gastrointestinal absorption and blood–brain barrier (BBB) permeability. The compound is predicted to be gastrointestinally absorbed while showing limited BBB penetration, suggesting potential safety advantages for anti-inflammatory drug development.

## DISCUSSION

### Phylogenetic analysis

Sequencing of the ITS (Internal Transcribed Spacer) rDNA from the fungal isolate (*Eurotium chevalieri*) confirmed its identity with a similarity of 99.80–100 and 100% query coverage to other *E. chevalieri* strains (accession no. PX498623) in GenBank, including type strain *E. chevalieri* ATCC 16,883 (accession no. NR111041). A high sequence identity firmly confirms the correct taxonomic classification of the isolate.

A phylogenetic tree constructed using closely related species retrieved from GenBank confirmed that the isolate was firmly positioned within the *E. chevalieri* clade. The inclusion of *Penicillium citrinum* as an outgroup provided a reliable external reference for rooting the tree, consistent with previous studies demonstrating the monophyletic nature of the genera *Aspergillus* and *Penicillium* [21, 22]. The ITS region is widely regarded as the universal DNA barcode for fungi due to its interspecific variability and conserved flanking regions [23]. The clear resolution of *E. chevalieri* within its clade further supports the effectiveness of ITS sequences in distinguishing even closely related xerophilic species [24–27]. Integrating molecular data with morphological and biochemical characteristics strengthens the identification process and offers robust support for ecological, industrial, and pharmacological investigations involving *Eurotium* and related taxa.

## GC-MS profile of *Eurotium chevalieri* (AUMC 16,390) extract

The GC–MS analysis of the ethyl acetate extract of *Eurotium chevalieri* AUMC 16,390 revealed a diverse array of metabolites, including esters, terpenoids, halogenated compounds, phenolics, and steroidal molecules. The predominant compound identified was Bis-(2-ethylhexyl) phthalate (BEHP), representing 19.179% of the extract. BEHP is a well-documented phthalate ester known for its broad-spectrum biological activities, including antimicrobial and larvicidal effects. For example, BEHP isolated from *Lactiplantibacillus plantarum* exhibited significant antibacterial activity against *Escherichia coli* and *Staphylococcus aureus*, in addition to potent larvicidal activity against *Culex quinquefasciatus*, achieving 100% mortality at 250 ppm after 72 h [28]. Similarly, BEHP produced by *Bacillus subtilis* demonstrated antimicrobial activity against several pathogens, including *Salmonella typhimurium* and methicillin-resistant *Staphylococcus aureus* (MRSA) [29]. Phthalate derivatives such as BEHP also possess diverse biological properties, including antifungal [30, 31], antitumor [32], antiretroviral [33], anticancer [34], antidiabetic [35], and antimalarial activities [36].

Another major compound detected was 7,7,8,8-tetramethylbicyclo[4.2.0]octa-1(6),3-diene-2,5-dione, accounting for 37.504% of the extract. Although specific studies on this compound are limited, its structural features suggest potential bioactivity, particularly cytotoxic and anti-inflammatory properties. Phenolic compounds are widely recognized as potent antioxidants or free-radical terminators due to their hydrogen-donating capacity [37, 38].

The extract also contained a high proportion of 12-Hydroxy-(5α,12β)-androstane-3,17-dione (35.788%), indicating the ability of *E. chevalieri* to produce steroidal compounds. Steroids are well known for their diverse biological functions, including anti-inflammatory and anticancer activities [39, 40].

The presence of di-tert-butylphenol (0.419%) further highlights the occurrence of phenolic molecules in the extract. Such compounds have been reported to exhibit antioxidant [41], anti-inflammatory [42], antimicrobial [43, 44], and antiviral effects [45]. 2,4-Di-tert-butylphenol (2,4-DTBP) is a natural lipophilic phenol identified in at least 169 organisms [46] and is known for its potent cytotoxicity. It exhibited notable cytotoxic activity against HeLa cells with an IC₅₀ value of 10 μg/mL [47] and induced apoptotic gene expression at levels comparable to cisplatin [48]. In the same study, 2,4-DTBP significantly upregulated *p53* and *caspase-7* expression in MCF-7 and A431 cell lines, with cell-line-specific differences in apoptotic response [48]. Additionally, 2,4-DTBP has been reported to possess adulticidal, larvicidal, ovicidal, repellent, and oviposition-deterrent activities against the spider mite *Tetranychus cinnabarinus* [49].

Low-abundance fluorinated compounds, including pentafluoropropionic acid, pentadecyl ester (0.480%), heptafluorobutyric acid, pentadecyl ester (0.801%), and pentafluoropropionic acid, dodecyl ester (0.392%), were detected in the GC–MS profile of the *Eurotium chevalieri* AUMC16390 extract. Naturally occurring fluorinated metabolites are considered rare in fungal secondary metabolism due to the limited environmental availability of fluorine and the absence of dedicated fluorination pathways in most fungi [10, 50]. Nevertheless, sporadic reports have described the presence of halogenated, and occasionally fluorinated, compounds in fungal extracts, typically at low abundance [51, 52]. Such compounds are often associated with enhanced biological activity, including antimicrobial effects, attributed to increased lipophilicity and membrane permeability. Accordingly, the fluorinated esters detected in this study are interpreted as minor constituents contributing to the overall chemical diversity of the extract rather than dominant or characteristic biosynthetic products of *E. chevalieri*.

Several oxygen-containing heterocycles were detected as well. Oxygen- and nitrogen-containing heterocyclic scaffolds such as phthalan, isochroman, and isoindoline are common in natural products and appear in diverse biologically active molecules, including antimycotics, antibiotics, antioxidants, pigments, and fluorophores. Their importance in medicinal chemistry is well established, as reflected by several top-selling pharmaceuticals—such as sofosbuvir, rosuvastatin, lenalidomide, aripiprazole, ibrutinib, and apixaban—that incorporate heterocyclic motifs [53]. Compounds containing a 1,3-dihydroisobenzofuran moiety, such as (12bS)-(+)-8,11-bihydroxy-12b-methyl-1 H-benzo [6, 7]phenanthro[10,1-bc]furan, are increasingly reported in nature. Pestacin, a representative molecule isolated from *Pestalotiopsis microspora* [54], exhibits strong antimycotic [55] and antioxidant properties, including potent DPPH radical-scavenging activity [56].

Eicosene (1.046%) and E-15-heptadecenal (1.393%) were also detected in the extract. Eicosene, found in essential oils and organic extracts of *Cestrum nocturnum*, displays activity against food-borne pathogens [57], contributes to the antimicrobial properties of *Allium atroviolaceum* flowers [58], and is a major component of *Aloe vera* extracts exhibiting strong antimicrobial activity [59]. Eicosene has also been reported to exert antifungal, antibacterial, antitumor, and cytotoxic activities [32, 60]. E-15-heptadecenal, an aldehyde present in *Spirulina platensis* extracts, has documented antibacterial activity [61].

The chemical profile of *E. chevalieri* underscores its potential as a rich source of bioactive metabolites with diverse therapeutic applications. The detection of compounds such as BEHP, steroidal metabolites, and halogenated esters aligns with previous reports describing the bioactive secondary metabolite repertoire of species within the genus *Eurotium* [62].

## Antimicrobial activity

The present study demonstrated that the crude extract of *Eurotium proliferans* AUMC 16,394 exhibited promising antimicrobial activity against a range of pathogenic microorganisms, including bacteria and fungi.

Of antibacterial activity, the extract showed noticeable inhibitory effects against both *Staphylococcus aureus* ATCC 25,923*, Bacillus cereus* ATCC 14,579 & *Bacillus subtilis* ATCC 6633 (Gram +ve) and *Escherichia coli* ATCC 25,922, *Klebsiella pneumoniae* ATCC 13,883, *Salmonella enterica* ATCC 14,028 & *Pseudomonas aeruginosa* ATCC 27,853 (Gram –ve). Interestingly, the inhibition zone was larger for *E. coli*, suggesting that the extract contains compounds capable of disrupting both Gram-positive and Gram-negative bacterial cells. This broad-spectrum activity may be attributed to secondary metabolites, which are commonly produced by *Eurotium* species.

Whereas, regarding antifungal potential, the extract was effective against *Candida albicans* and *C. tropicalis* due to structural variations in the fungal cell wall or membrane composition, which influence the permeability and binding of antifungal compounds.

Previous reports have confirmed that *Eurotium* species produce a wide range of secondary metabolites with antimicrobial and other pharmacological properties. For example, Asperentin, isolated from *Eurotium repens*, has demonstrated significant antibacterial, antifungal, and cytotoxic activities [63]. Additionally, flavoglaucin and isoterrahydroauroglaucin from marine *Eurotium* spp. were found to exhibit anti-inflammatory and antimicrobial effects through the inhibition of pro-inflammatory mediators [64]. The antimicrobial activity reported in this study is based on agar well and disc diffusion assays, which are widely used for preliminary screening of bioactive extracts. However, these methods are inherently semi-quantitative, as inhibition zone diameters can be influenced by compound diffusion properties, molecular size, and agar composition. Consequently, while the observed inhibition zones indicate promising antimicrobial potential, minimum inhibitory concentration (MIC) and minimum bactericidal/fungicidal concentration (MBC/MFC) assays will be necessary in future investigations to provide a more precise and quantitative evaluation of antimicrobial efficacy.

## Molecular docking

### Secondary metabolites as inhibitors of enoyl-[acyl carrier protein] reductase (FabI)

Interpreting the FabI docking results in terms of both binding affinity and metabolite abundance gives them biological significance. Despite having the highest predicted FabI binding affinity (−8.171 kcal/mol), the low relative abundance of **compound 1** (1.362%) indicates a limited contribution to the crude extract’s overall antibacterial activity. Similarly, despite forming stabilizing hydrogen bonds with Gly93A and Ile192A, **compound 2**, which is present at trace levels (0.419%), is unlikely to play a significant role.

On the other hand, **compound 4** (37.5%) and **compound 3** (35.8%) combine high abundance with FabI docking scores similar to the co-crystallized inhibitor triclosan. Crucially, both substances interact with residues that are essential for FabI catalysis and inhibitor recognition, such as Lys163A, Tyr156A, Thr194A, and Ile200A, which triclosan also engages. A common inhibitory mechanism involving disruption of bacterial fatty acid biosynthesis, which ultimately hinders membrane formation and bacterial growth, is supported by these interaction patterns [65]. All things considered, this analysis suggests that these two dominant metabolites, rather than lower-abundance compounds with higher individual docking scores, are most likely responsible for the antibacterial activity of the E. chevalieri AUMC16390 extract.

### 2- Potential Anti-Inflammatory Activity of the Fungal Steroid Revealed by Glucocorticoid Receptor Docking:

Molecular docking analysis suggests that 12-hydroxy-(5α,12β)-androstane-3,17-dione may function as a natural modulator of the glucocorticoid receptor (GR). The compound adopts a binding orientation closely resembling that of the co-crystallized ligand dexamethasone, with a calculated RMSD value of 2.085 Å, indicating compatibility with the GR ligand-binding pocket. Stabilization of the docked complex is supported by a hydrogen bond interaction with Arg611A, along with hydrophobic contacts involving Met604A and Met646A, residues known to contribute to ligand recognition and receptor activation [66].

Given the central role of GR activation in suppressing inflammatory signaling pathways through transrepression of NF-κB and AP-1, these interactions provide a plausible molecular explanation for the observed anti-inflammatory potential of the fungal extract. However, it must be noted that functional agonism cannot be conclusively inferred from docking studies alone, and experimental validation is required to confirm receptor activation.

Importantly, while docking and in silico analyses support the biological relevance of this steroid-like metabolite, its structural assignment remains putative. Comprehensive characterization using 1D/2D NMR and HRMS will be necessary in future studies to unequivocally confirm the proposed structure and establish structure–activity relationships.

## Conclusions

The chemical profiling of the ethyl acetate extract from *Eurotium chevalieri* (AUMC 16,390) using GC–MS revealed a complex mixture of structurally diverse metabolites, including esters, diketones, alkenes, furan and halogenated compounds. The predominance of diketones and phthalate derivatives, as well as the presence of fluorinated esters and substituted phenols, indicates the potential biological significance of the extract. These findings support the role of *E. chevalieri* as a valuable source of bioactive compounds with possible antimicrobial, antioxidant, and pharmacological properties. The results serve as a foundation for future studies aimed at isolating and characterizing these compounds in greater detail, as well as assessing their specific biological activities in vitro and in vivo. Further research may also explore the biosynthetic pathways responsible for producing these metabolites, which could enhance their application in pharmaceutical and industrial biotechnology.

## Data Availability

The ITS sequence obtained in this study has been deposited in GenBank under accession number PX498623. The record is available for peer-review access. All other data generated or analyzed during this study are included in this published article and its supplementary information files.
